# Effect of Lignin Modifier on Engineering Performance of Bituminous Binder and Mixture

**DOI:** 10.3390/polym13071083

**Published:** 2021-03-29

**Authors:** Chi Xu, Duanyi Wang, Shaowei Zhang, Enbei Guo, Haoyang Luo, Zeyu Zhang, Huayang Yu

**Affiliations:** 1 School of Civil Engineering and Transportation, South China University of Technology, Wushan Road, Tianhe District, Guangzhou 510000, China; x.c07@mail.scut.edu.cn (C.X.); tcdywang@scut.edu.cn (D.W.); 201830120092@mail.scut.edu.cn (E.G.); 201864120167@mail.scut.edu.cn (H.L.); huayangyu@scut.edu.cn (H.Y.); 2 Institute of Highway Engineering, RWTH Aachen University, Mies-van-der-Rohe-Street 1, 52074 Aachen, Germany; zeyu.zhang@isac.rwth-aachen.de

**Keywords:** lignin, bituminous modifier, lignin modified bitumen, chemical analysis, rheological behavior, mechanical properties

## Abstract

Lignin accounts for approximately 30% of the weight of herbaceous biomass. Utilizing lignin in asphalt pavement industry could enhance the performance of pavement while balancing the construction cost. This study aims to evaluate the feasibility of utilizing lignin as a bitumen performance improver. For this purpose, lignin derived from aspen wood chips (labeled as KL) and corn stalk residues (labeled as CL) were selected to prepare the lignin modified bituminous binder. The properties of the lignin modified binder were investigated through rheological, mechanical and chemical tests. The multiple stress creep recovery (MSCR) test results indicated that adding lignin decreased the J_nr_ of based binder by a range of 8% to 23% depending on the stress and lignin type. Lignin showed a positive effect on the low temperature performance of asphalt binder, because at −18 °C, KL and CL were able to reduce the stiffness of base binder from 441 MPa to 369 MPa and 378 MPa, respectively. However, lignin was found to deteriorate the fatigue life and workability of base binder up to 30% and 126%. With bituminous mixture, application of lignin modifiers improved the Marshall Stability and moisture resistance of base mixture up to 21% and 13%, respectively. Although, adding lignin modifiers decreased the molecular weight of asphalt binder according to the gel permeation chromatography (GPC) test results. The Fourier-transform infrared spectroscopy (FTIR) test results did not report detectable changes in functional group of based binder.

## 1. Introduction

Lignin is a typical biopolymer of lignocellulosic biomass, which is abundantly generated in paper making and biofuel industry. It accounts for approximately 30% of the weight of herbaceous biomass [1]. The chemical nature of lignin is a hydrocarbon consisting of benzene ring, which are connected by methoxy groups, carbonyl groups and aliphatic double bonds randomly [2]. Lignin consists of plentiful aromatic rings attached with alkyl chains. It is a highly branched and amorphous biomacromolecule with the average molecular weight in the range of 1000 to 20,000 g/mol, depending on the production process. Although it is known as the second most abundant biopolymer around the world, the traditional application of lignin is mostly limited to fuel, while only a small amount of lignin has been used as value-added bioproducts [3]. With the features of well-sources and high content of aromatic structures, lignin is an underlying green bio-resource which can be utilized as a modifier to substitute for other industrial aromatic polymers. For instance, it is potential to be utilized as bitumen modifier on pavement engineering for better engineering performance and cost saving.

**Figure 1:**
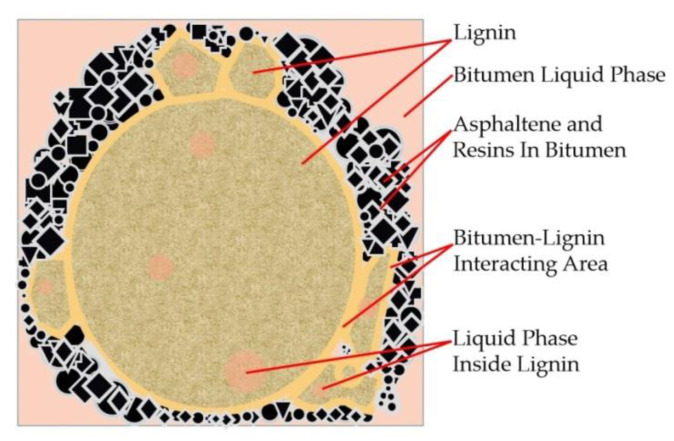
Schematic of the mechanism of the bitumen-lignin working system.

As a by-product of the petroleum refining, bitumen has been extensively applied as gluing binder to bond the loose aggregates for pavement construction [4]. Bituminous pavement has been extensively accepted and largely convincing because of its attractive advantages including improved smoothness, low traffic noise and easy maintenance. As the rheological properties of bitumen largely determine the performance of the pavement, a series of bitumen modifiers have been developed and applied to improve the durability of pavement. Bitumen modifiers reduce the temperature sensitivity of asphalt binder, making it harder in evaluated temperate and softer in low temperature condition, thus enhancing the rheological behavior as well as the service life. The schematic of the mechanism of the bitumen-lignin working system is shown in Figure 1. The incorporation of lignin modifier results in the absorption of bitumen liquid phase into the bitumen-lignin interacting area during the mixing process, which forms the bitumen-lignin working system and changing the viscoelastic behavior of bitumen binders. Conventional bitumen modifiers include styrene-butadiene-styrene (SBS polymer [5,6], crumb rubber [7,8], bio oil [9], plastics [10] and various fibers [11,12]. The main components of raw bitumen are statures, aromatics, resins and asphaltenes, which have compatibility with the above-mentioned modifiers. For example, SBS modifier forms the swallowed modifier network in asphalt fraction and makes the bituminous fractions more viscous [13]. Crumb rubber enhances the engineering performance by both the polymer modification effect of soluble components and the particle effect of insoluble particles [14]. However, the use of bitumen modifiers increase the material cost of asphalt pavement construction [15]. As the second most abundant biopolymer around the world, lignin is fully adapted to the requirements of large-scale applications in bitumen pavements with limited additional expense [16]. Therefore, the application of different types of lignin as bitumen modifier has been a hot research topic for pavement researchers.

Previous researches have shown encouraging findings on the application of lignin as bitumen modifier. It is now well established that the performance of modified bitumen materials largely depended on the types of lignin. Xu et al. evaluated the feasibility of the application of lignin as a substitute for bituminous binder by rheological method. The results also demonstrated that lignin can improve the stiffness and rutting resistance in bituminous binder without deteriorating other properties [17]. In McCready and Williams’s study, it was proven that lignin can improve the temperature sensitivity of raw bitumen [18]. Pan found that lignin delayed the ageing rate of bitumen [19]. Batista et al. showed that bituminous binder will be superior in both rutting and cracking resistance after modification. The incorporation of lignin also improve thermal stability of bitumen [20]. Arafat et al. used three different types of lignin for asphalt modification, and obtained a significant improvement in rutting resistance, cracking resistance, and moisture damage susceptibility [21]. In the study of Xie and coauthors, the feasibility of lignin as a sustainable bitumen modifier were demonstrated in terms of engineering and economic [22]. Gao et al. found that the incorporation of lignin from waste wood chips reduced the fatigue life of the bitumen, but the reduction was small when the content of lignin was below 8% [23]. Norgbey et al. reported that the addition of 10% lignin form corncobs insignificantly influenced the workability and compactability of the mixture [16].

Although plenty of studies have demonstrated the feasibility of utilizing lignin as a bitumen modifier, the application of the Kraft lignin (KL) and the corn stalk lignin (CL) as bitumen modifier are quite limited. Both KL (25 million tons/year) and CL (250 million tons/year) have abundant source from paper producing industries and agricultural productions [20,24], respectively. To date, the performance of lignin modified bituminous binder and mixture have still not been comprehensively investigated. Hence, this study was conducted to obtain a more comprehensive understanding of lignin modified bituminous binder and mixture by a series of experimental tests. To achieve this goal, rheological tests including Superpave performance grading test [25], frequency sweep test [26], multiple stress creep recovery(MSCR) test [27], liner amplitude sweep test [28], gel permeation chromatography test [29], and Fourier-transform infrared spectroscopy test [30] were performed on lignin modified asphalt (LMA) binders. Moreover, corresponding mechanical properties including Marshall Stability [31], aging resistance [32], and moisture susceptibility [33] were tested. It is expected that this paper can provide helpful information regarding to sustainable lignin-based alternatives for pavement materials.

## 2. Materials and Methods

### 2.1. Raw Materials and Preparation of Sample

#### 2.1.1. Materials

Bitumen with penetration value range from 60 to 70 (shortly named as Pen60/70) was used as virgin binder in this research [34]. Pen60/70 was supplied by Guangzhou Xinyue Transportation Technology Co. Ltd., Guangdong, China. Two different lignin powders (with size less than 100 mesh) were used to modify virgin bitumen by wet process. They are Kraft lignin (KL) from Nanjing Dulai Biotechnology Co., Ltd. (Nanjing, China) and corn stalk lignin (CL) from Jinan Yanghai Environment Materials Co., Ltd (Jinan, China). They were passed through a #100 sieve, i.e., the size of lignin power is below 0.15 mm. Their properties are given in Table 1.The Kraft lignin (KL) powder in the brown was derived from aspen wood chips. Another one, the yellow lignin power was extracted from the corn stalk residues. This lignin was labeled as CL. The figures gathered by the scanning electron microscope (SEM) were shown in Table 1. It can be observed that KL had a relatively clear hemispherical hollow shell structure. In comparison, CL presented a loose powdery structure under the electron microscope due to its poor conductivity.

The diabase aggregates and mineral powder were selected. Then the aggregates were subjected to washing, drying, and sieving to satisfy the grading requirement. The stone matrix asphalt mixture with 10-mm nominal maximum aggregate size (SMA10) was selected as the gradation for the preparation of asphaltic mixture, which is widely used in south China. The designed gradation information is shown in Table 2.

#### 2.1.2. Sample Preparation

Two lignin modified asphalt binders were prepared in this research, KL modified asphalt binder (labeled as KLA) and CL modified asphalt binder (labeled as CLA). They were prepared by mixing KL and CL modifiers (5% by weight Pen60/70) with virgin asphalt, respectively. A high-shear radial flow impeller was used to mix the virgin asphalt binder and modifiers. All blended mixes were prepared at the temperature of 160 °C for one hour. The mixing speed of 4000 rpm was selected in this study. 

All unaged asphalt binder samples (including modified and virgin asphalt binders) were aged under different ageing processes. According to different aging conditions, the aging degree can be divided into three types: unaged, short-term aged and long-term aged. In this study, the short-term aging process of asphalt binder was achieved through the rolling thin-film oven (RTFO) method in line with AASHTO T240 [35]. Then, the short-term aged samples were exposed to the long term aging through the pressure aging vessel (PAV) test according to AASHTO R28 [36]. In the PAV test, this research simulated the aging of the asphalt binder after 10 years of used on the actual road surface. To remove moisture before being mixed with hot asphalt binder, the pre-treated diabase stones and mineral powder were placed in an oven for more than 4 h with the temperature 180 °C. The optimum asphalt content was determined based on JTG F40-2004 [37]. The final determined contents were 4.5% identically. The blending temperature of asphalt and diabase stones and the preparing temperature of KLA and CLA were set at 160 °C. The compacting temperature was set at 140 °C. As AASHTO and ASTM requires, 4% target air void for specimens in Marshall test [31] and the Indirect Tensile Stiffness Modulus (ITSM) [32] test, whereas 7% in Indirect Tensile Strength (ITS) test [33].

### 2.2. Methods

#### 2.2.1. Rheological Tests

##### Penetration and Softening Point Test

The empirical properties of the binder were assessed by the penetration test [38] and softening point test [39]. The consistency of bitumen binders was estimated through penetration test. According to test specification, it depends on the depth of the needle (100 ± 0.1 g) penetrating a standard bitumen sample under the condition of 5 s and 25 °C. A steel ball weighting 3.5 g was put on the surface of the formed binder during the softening point test. The test temperature increased at a constant rate until the steel ball dropped out, and then the temperature, which was an index to assess the high-temperature performance, was recorded to be the softening point of bitumen binder. 

##### Rotational Viscosity Test

The workability of bitumen binder can be characterized by measuring its rotational viscosity. The test was measured at 135 °C and 160 °C. 135 °C is the conventional viscosity testing temperature required by AASHTO PG testing standards [40], and 160 °C is the common testing temperature for polymer modified asphalt due to its higher viscosity. All test procedures are in compliance with AASHTO T316 [40]. A Brookfield rotational viscometer was employed to evaluate the rheological properties of bitumen binder. It was noted that the types of asphalt binder should matched to the sized of spindles.

##### Rutting Parameter Test

The rheological properties of bitumen binder can be obtained by conducting the Rutting Parameter test [26]. In this test, a dynamic shear rheometer (Malvern Kinexus Lab+, Malvern analytical Company, Malvern, UK) was utilized. The rutting parameter was the characterization of the high-temperature performance of all types of binders. The complex shear modulus (G*) and phase angle (δ) were used to obtain rutting parameter G*/sinδ. Unaged and RTFO-aged bitumen binders were prepared for the rutting parameter test with a plate 25 mm diameter and a 1 mm plate gap. The rutting parameter G*/sinδ test begun with 64 °C with an interval of 6 °C, then temperature automatically increased until the obtained rutting parameter was smaller than the critical number detailed in AASHTO T315 [26], i.e., 1.0 kPa for unaged asphalt binders and 2.2 kPa for RTFO-aged asphalt binders.

##### Fatigue Parameter Test

The rheological properties of bitumen binder can be determined by conducted the Fatigue Parameter (G*sinδ) test [26]. The fatigue parameter characterizes the intermediate temperature performance of all kinds of binders. In this test, a dynamic shear rheometer (Malvern Kinexus Lab+, Malvern analytical Company, Malvern, UK) was used. PAV aged asphalt binders were prepared for the fatigue parameter test with a plate 8 mm diameter and a 2 mm plate gap. The fatigue parameter test begun from 28 °C and have an increasing gap of 3 °C until the fatigue parameter exceeded 5000 kPa.

##### Bending Beam Rheometer (BBR) Test

The low-temperature performance of all types of binders can be estimated by conducted the BBR test. Two important parameters the stiffness and m-value, were used to estimate the low-temperature thermal cracking resistance according to AASHTO T313 [41]. The bitumen binder after PAV procedures was prepared for the BBR test [41]. The BBR test was conducted in a temperature fluid bath at constant load of 980 ± 50 mN and 240 s, and test temperatures started at −6 °C with a decrement of 6 °C. Moreover, The BBR test was complied with AASHTO T313, a small asphalt beam specimen was made to simulate the stress applied in pavement structure in a low-temperature environment. 

##### Multiple Stress Creep Recovery (MSCR) Test

The MSCR test was performed to quantify the resistance of bitumen binder to permanent deformation according to AASHTO T350 [27]. The test temperature was set at 60 °C. R% is a parameter that presents the average percent recovery, J_nr_ is another parameter that expresses the irreversible creep compliance and J_nr-diff_ is a parameter that evaluates the stress sensitivity calculated at both stress levels. R%, J_n_r and J_nr-diff_ were the chosen parameters to value the recoverable and non-recoverable deformation of bitumen binders. Following AASHTO MP19 [27], a creep load was implemented to test samples for 1 s and then recovered for 9 s under unloading condition. Creep and recovery cycles were implemented for 10 cycles at the lower stress level (0.1 kpa), followed by another 10 cycles at the higher stress level (3.2 kPa) Superior resistance to permanent deformation is associated with lower J_nr_, while the stress sensitivity was evaluated by J_nr-diff_. The R% and J_nr_ parameters were calculated by following equations [42].

(1) $$R\%=\frac{\varepsilon_{m}-\varepsilon_{\mathrm{nr}}}{\varepsilon_{p}}$$

(2) $${\;J}_{\mathrm{nr}}=\frac{\varepsilon_{\mathrm{nr}}}{\sigma }$$

where ε_m_/ε_nr_/ε_p_ is the maximum/non-recoverable/percentage strain; σ is the stress level, 0.1/3.2 kPa.

##### Linear Amplitude Sweep (LAS) Test

The Linear Amplitude Sweep (LAS) test was performed to calculate the anti-fatigue damage capacity of bitumen binders. The PAV-aged bitumen binders were prepared and the test temperature was 25 °C. The test procedures were completely in compliance with AASHTO TP101-14 [28]. In the LAS test, the first step was performed using the frequency sweep test, followed by linear amplitude strain sweep. During the frequency sweep test, a strain level of 0.1% was applied at a frequency range of 0.2–30 Hz. Linear amplitude sweep test was performed at a frequency of 10 Hz within the range of 0–30% strain after the frequency sweep test was completed. The viscoelastic continuous damage (VECD) method was employed to calculate the value of cycles to failure at 2.5% and 5% strain levels. Finally, the fatigue resistance of the sample can be represented by the number of cycles to failure (N_f_). The N_f_ was calculated using the following equation.

$$N_{f}=A{\left(\gamma \right)}^{B}$$

where A is the VECD model coefficient; B = 2a (a is the fitting coefficients); γ is the applied strain (2.5% and 5%).

##### Frequency Sweep Test

Except for the above tests, the virgin and modified binders were also swept at different temperatures and frequencies to evaluate their overall rheological properties. According to the principle of time-temperature superposition, the reference temperature was set at 60 °C. To assess the overall rheological performance of test binders, a master curve of G* was recorded. A series of sweeps were performed at frequencies from 30 to 0.01 Hz over a range of 4 to 76 °C with a 12 °C gap. Based on Williams–Landel–Ferry (WLF) equation, the test data were matched to the best, and then the single master curve was obtained [9,43]. 

#### 2.2.2. Chemical Tests

##### Gel Permeation Chromatography (GPC) Test

The gel permeation chromatography (GPC) test was performed to analyze the molecular weight distribution of test samples [14]. A P230 Elite GPC with two chromatographic columns (PLgel 5 lm 103 + Å PLgel 3 lm Mixed-3) was utilized to segregate the constituents of the bitumen binders according to molecular size. The test samples were melted in Tetrahydrofuran (THF), and the THF-sample solution was drained through the chromatographic columns. The flow rate of injection was restrained (0.5 mL/min) as well as the chromatographic column temperature was also controlled at 40 °C. The percolation sequence of molecules in the GPC column was from the large molecules to small molecules. The concentrations of components were recorded using a refractive index differential (RID) detector, and to obtain the chromatogram consequently. Then the molecular size distribution was obtained by analyzing the chromatogram.

##### Fourier-Transform Infrared Spectroscopy (FTIR) Test

The FTIR test was conducted to analyze the chemical bonds of test samples. The instrument, Bruker Vertex 70 (Billerica, MA, USA) was used in FTIR test. Since each chemical functional group owns Special infrared ray absorption characteristics, the spectrum was measured by using the FTIR technique, and then the measured spectrum was compared with the known spectrum so as to analyze the chemical bonds of test samples [44]. It has been proven that FTIR is a useful probe to evaluate the chemical bonds and functional groups within asphalt material [45,46]. In the FTIR test, an FTIR spectrometer and pellets with a thickness of around 1 mm were used to scan the test samples and to gain the required infrared spectroscopy ranging. 

#### 2.2.3. Mechanical Property Tests

##### Marshall Test of Stability and Flow Value

The resistance of bituminous mixtures to deformation can be evaluate through Marshall test [31]. In the test, two parameters, namely Marshall Stability and flow value, were measured. The maximum experimental force that the specimen can bear under a specified loading condition with a constant speed (50 mm/min) is termed as Marshall Stability. Meanwhile, flow value is defined as the sum of deformed accumulation of the specimens when failure to resist. To assess the resistance to water damage, the specimens were placed in a constant temperature water bath, the test temperature was set at 60 °C and the experimental time was set at 30 min or 48 h. The residual Marshall stability (RS) was defined as the ratio of Marshall Stability of the specimen to the virgin specimen after soaking in hot water for 48 h. The higher value of RS indicates better moisture stability.

##### Moisture Susceptibility Test

The indirect tensile strength (ITS) test [33] was performed to calculate the moisture susceptibility of test specimens. During this test, the ratio between the indirect tensile strength after moisture adjustment and before moisture adjustment was employed to characterize the moisture susceptibility of tested specimens. To make a comparison, every kind of asphalt mixture specimen was divided into two groups, the control one and the freeze-thaw one. The control group was tested before any moisture adjustment; however, the freeze-thaw group was to make water saturated by a vacuum pump firstly, followed by a freeze-thaw cycle. During the freeze phase, the test specimens were placed at the condition of −18 °C for 16 h following by a thawing phase, the specimens were put in a thermostatic water bath at the condition of 60 °C for 24 h. After that, water bath in the condition of 25 °C for 2 h was applied to those specimens. Finally, the ITS test was conducted. A vertical loading with a specified loading rate (51 mm/min) was pressured by a circular arc with a certain width until the end of the fracturing of test specimens. 

##### Indirect Tensile Stiffness Modulus Test (ITSM)

The ITSM test [32] was performed to analyze the stiffness of mixtures. ITSM Ratio (ITSMR) was employed to reflect the aging sensitivity of test specimens, which was calculated after and before the long-term aging. In the test, the control group and the long-term aging group were set and each group of ITSM was acquired at 20 °C and 30 °C. The long-term aging group was put in a constant temperature oven (85 °C) for the five-day aging process, mimicking the aging conditions of a pavement after the construction of five to ten years of service life. ITSM Ratio (ITSMR) was employed as an indicator of the aging sensitivity of test samples, ITSMR equals to the ratio of the ITSM after and before the long-term aging and was calculated by following equation [47].

(3) $$\mathrm{ITSMR}=\frac{{\mathrm{ITSM}}_{\mathrm{after}\;\mathrm{aging}}}{{\mathrm{ITSM}}_{\mathrm{before}\;\mathrm{aging}}}$$

where ITSM_after aging_ is the ITSM after the long-term aging and ITSM_before aging_ is the ITSM before the long-term aging.

## 3. Results and Discussion

### 3.1. Rheological Tests

#### 3.1.1. Softening Point and Penetration

**Figure 2:**
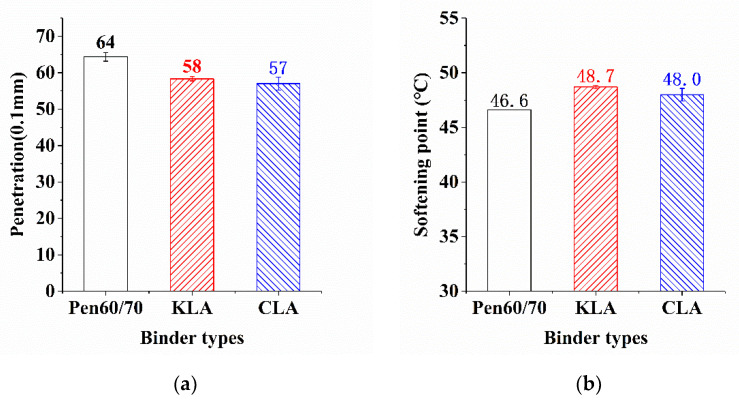
Results analysis (**a**) penetration and (**b**) softening point.

Figure 2 illustrates the consequences of penetration test (a) and softening point test (b). As the bar chart shows, it was observed that the application of lignin brought lower penetration values. The sink depths of the binders were measured by the penetration test. The lower penetration value indicates higher stiffness. It is clear from the chart that LMA reduced the penetration value of Pen60/70 regardless of lignin type, the penetration values of KLA and CLA decreased from 64 to 58 and 57 (0.1 mm), respectively. This result is consistent with the previous finding [20], the 5 wt.% of lignin addition caused a decrease in penetration. In contrast, higher softening points brought by the application of lignin were observed. This result is also similar to past studies [16]. The softening point values of KLA and CLA were 2.1 °C and 1.4 °C higher than Pen60/70, respectively. By comparison, LMA owned the lower penetration values and higher softening points showed superior performance in high service temperature. KLA behaved similarly to CLA, while KL had a slight improvement in high-temperature performance than that of CL.

#### 3.1.2. Workability

**Figure 3:**
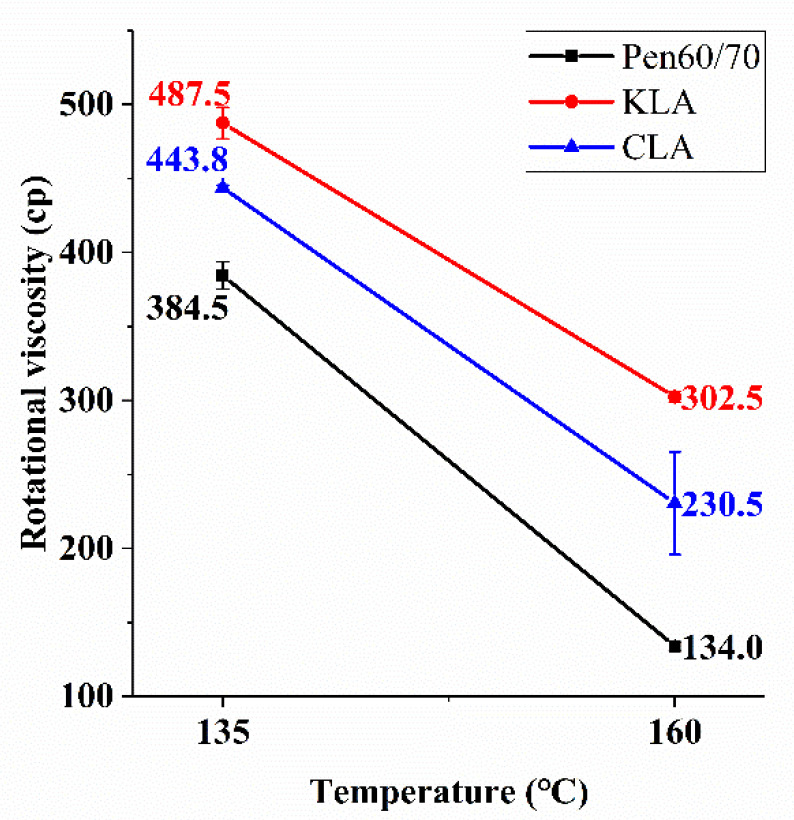
Rotational viscosity test results.

The viscosity values of the modified binder with 5 wt.% of lignin at temperatures of 135 °C and 160 °C are described in Figure 3. As a critical and widely used parameter, viscosity value can evaluate the mixability and workability of bitumen binders. In order to ensure adequate liquidity, the appropriate viscosity with good workability was necessary. As expected, the viscosity of test samples increased with the decreased temperature. The viscosity of Pen60/70, KLA, and CLA at 135 °C was 384.5, 487.5, and 443.8 cp, respectively. It is apparent from the line graph that all viscosity results meet the specifications of the requirement of AASHTO specification (i.e., 3000 cp). For construction purpose, all the bitumen binders have sufficient fluidity to be pumped. Same as previous studies, the application of lignin raised the viscosity of asphalt binders [20]. As depicted in Figure 3, at 160 °C, the viscosity of Pen60/70, KLA, and CLA was 134, 302.5, and 230.5 cp, respectively. It can be found that the viscosity of LMA is higher at all temperatures than that of Pen60/70. What’s more, KLA has the highest viscosity value, followed by CLA. At 160 °C, KLA has a viscosity value 2.2 times larger than the value of Pen60/70, and the viscosity value of CLA is 1.7 times larger than that of Pen60/70.

#### 3.1.3. Rutting Resistance

**Figure 4:**
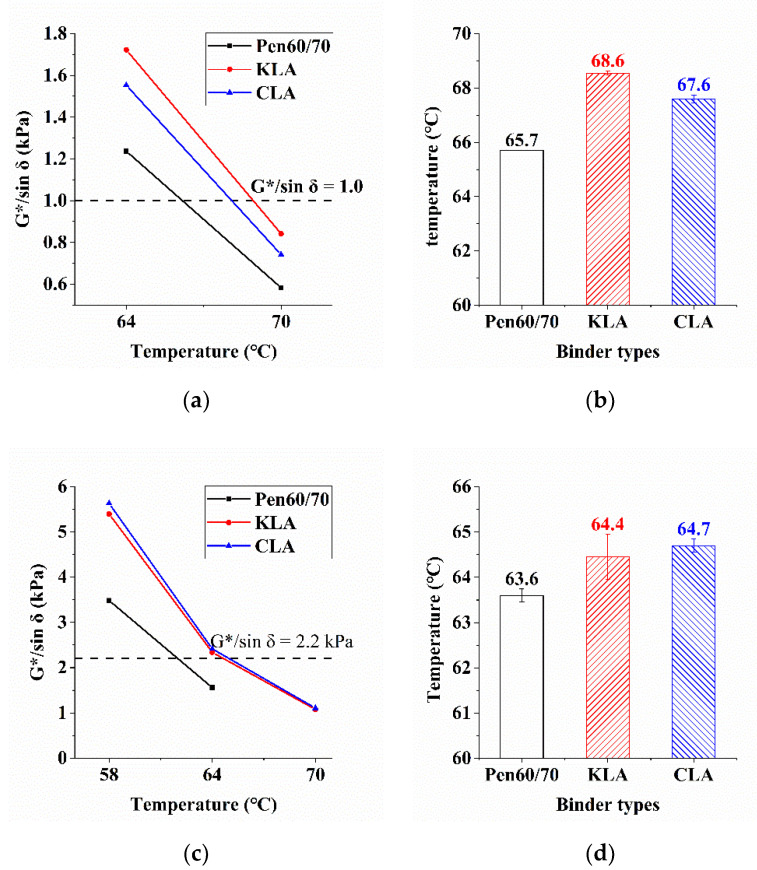
Superpave rutting parameter test results: (**a**) rutting parameter (unaged); (**b**) failure temperature (unaged); (**c**) rutting parameter (RTFO-aged) and (**d**) failure temperature (RTFO-aged).

The rutting resistance of bitumen binders are evaluated using the parameter G*/sinδ. The higher G*/sinδ is, the greater rutting resistance the bitumen binder has. Unaged and RTFO-aged, virgin and modified bitumen binders were tested. Figure 4a and c exhibit the testing temperature and the corresponding G*/sinδ value. The final failure temperatures (1.0 kPa for unaged asphalt and 2.2 kPa for short-term aged asphalt) are plotted in Figure 4b and d. As shown, with the application of lignin, the higher value of rutting factor and failure temperature of Pen60/70 were obtained regardless of the sources of lignin. When 5 wt.% of lignin was added, KLA contributed the highest improvement in performance at high failure temperature (68 °C), followed by CLA (67.6 °C). The G*/sinδ values of RTFO-aged binders were evaluated at 58 °C, 64 °C, and 70 °C. The results make it clear that adding lignin increased the G*/sinδ values regardless of aging stages. The results are similar to the past study conducted by Xu [17]. The addition of lignin promoted the high-temperature grade of RTFO-aged samples regardless of the lignin sources. By contrast, the difference between KLA and CLA was not prominent in short-term aged condition, and unaged KLA owned slightly stronger resistance to rutting at the high temperature.

The MSCR test was also implemented to value the binder performance at high temperature, the recovery and non-recovery characters of the RTFO-aged binders were analyzed with the stress levels of 0.1 kPa and 3.2 kPa. The J_nr_ and R% are exhibited in Table 3. The maximum difference of J_nr_ is no more than 8%, which is obviously in compliance with the specification of AASHTO MP19 (<75%). As the stress level increased, all R% values decreased while J_nr_ values increased. Table 3 shows that the unmodified binder did not have any recoverable portion at 3.2 kPa stress level at 64 °C, while the recovery ratio of lignin modified binders at 3.2 kPa stress level had 0.1% (KLA) and 0.2% (CLA), respectively. By comparison, the virgin bitumen owned the highest J_nr_ values at both stress level, lignin modified binders owned close J_nr_ values, showing moderate improvement in the resistance to rutting. The results were consistent with those obtained by softening point test as well as G*/sinδ. Similar results were found by Arafat [21]. Furthermore, CLA possessed the lowest J_nr_ values at both stress levels and highest R% value at high-stress level, proving that the application of CL resulted in more elastic behavior of asphalt binder. KLA possessed highest R% value at low-stress level, which indicated that adding KL led to more deformations recovered.

#### 3.1.4. Fatigue Resistance

**Figure 5:**
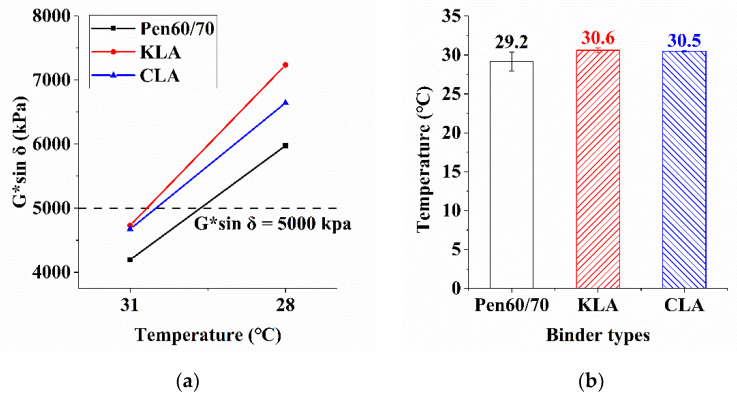
Results analysis: (**a**) fatigue parameter (PAV-aged) and (**b**) failure temperatures (PAV-aged).

The fatigue resistance of bitumen binders was investigated by the LAS test and the evaluation indicators is G*sinδ. Figure 5a shows the connection between test temperatures and G*sinδ values. Figure 5b provides the corresponding failure temperatures when the G*sinδ value was equal to 5 MPa specified by AASHTO M320. As shown in Figure 5a, as test temperature decreased, the G*sinδ values of all kinds of bitumen binders increased. Both at 31 °C and 28 °C, the G*sinδ values of KLA were the highest, that of Pen60/70 was the lowest. The fatigue resistance of the test binder will deteriorate with the increase of the fatigue failure temperature [9]. Figure 5b presents the failure temperatures, it can be observed that the failure temperature of KLA was 30.6 °C, the highest value of the three bitumen binders, followed by the CLA (30.5 °C). The virgin asphalt without lignin had the lowest failure temperature, which indicated the modest negative effect on the resistance of bitumen binders to fatigue brought by the application of lignin. The effect of lignin modifier on fatigue performance is negative. However, the negative effect is not significant. At 31 °C, the addition of KL and CL increased the G*sinδ value by 12.7% and 11.4% respectively. While at 28 °C, the addition of KL and CL increased the G*sinδ value by 21.1% and 11.2% respectively. In terms of failure temperature, it is noted that KL and CL modified asphalt have 1.4 °C and 1.3 °C higher than virgin binder, indicating poorer fatigue resistance. The findings support previous works by Xu and Norgbey [16,17]. By comparison, The KL further enhanced the negative effect by increasing the temperatures by 1.4 °C, 0.1 °C higher than that of CL.

**Figure 6:**
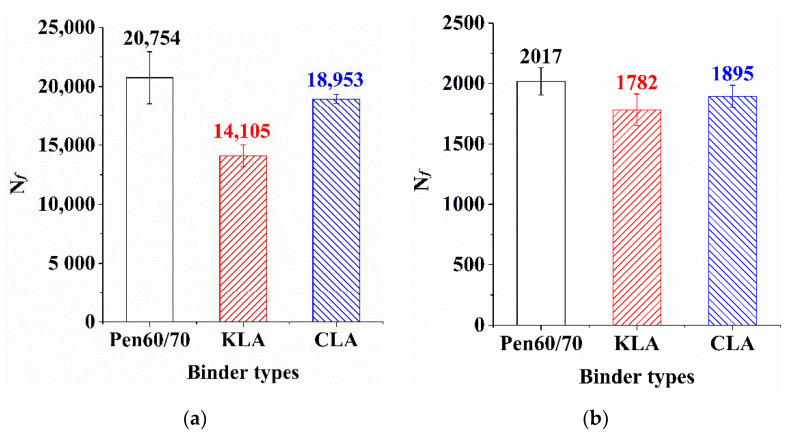
LAS test results analysis: (**a**) strain value 2.5% and (**b**) strain value 5.0%.

The fatigue performance of bitumen binders was measured through LAS test using the PAV-aged samples. The results at two strains levels (2.5% and 5%) are presented in Figure 6. The fatigue life (N_ƒ_) results reflect that the high strain applied to the bitumen binders reduced the fatigue life regardless of whether the lignin was used and whatever the sources of lignin were. The higher value of the cycles to fatigue (N_ƒ_) is, the worse fatigue resistance the test binder owns [47]. According to Figure 6, Pen60/70 was responsible for the higher cycles and the application of lignin reduced the values of N_ƒ_ at both strain levels. By comparing with Pen60/70, the N_ƒ_ value of KLA was smallest at two strains levels, followed by CLA. The N_ƒ_ value of KLA decreased by 11.65% at the strain level of 2.5% and 32.04% at the strain level of 5%, the N_ƒ_ value of CLA decreased by 6.05% at the strain level of 2.5% and 8.68% at the strain level of 5%. The results indicate that the incorporation of lignin modifiers brought the reduction of fatigue life to the test binders. The similar LAS result on the negative impact of lignin on the fatigue resistance of bitumen binders was also found by other studies [16,17]. This is because the application of lignin can make the asphalt binder hard [17]. In addition, compared to KL, CL possessed better fatigue resistance performance. 

#### 3.1.5. Low Temperature Performance

The BBR test was done to analyze the low-temperature properties of test binders at three low temperatures (−6, −12 and −18 °C). Two parameters, stiffness and the creep rate (m-value) of test binders, were showed in Table 4. Binders with higher m-value and lower stiffness provided better performance in low-temperature condition. Table 4 shows the stiffness of all binders are less than 300 MPa and the m-value are greater than 0.3 at −6 and −12 °C, which conforms to AASHTO T313. Compared to Pen60/70, it was observed that the LMA binders owned lower stiffness and greater m-value. Compared to KLA, the m-value of CLA was slightly larger and the stiffness was smaller except −18 °C. Thus, LMA showed a slightly better low-temperature performance than raw binder, the low-temperature properties of CLA was slightly better than KLA. The BBR test results were inconsistent with previous study that adding lignin into bitumen binder had little negative effect on thermal cracking potential [17]. However, the finding was also supported by other study that lignin binder had higher resistance to thermal cracking at low temperatures [20].

#### 3.1.6. Overall Rheological Behavior

The test binders were swept at different temperatures (4–76 °C) and a series of frequencies (30–0.01 Hz) in the frequency sweep test. Then master curves of G* at 60 °C within a broad frequency angle (10^−3^–10^−7^ Hz) were depicted according to the principle of time-temperature superposition. To get the master curves, many complicated calculations were performed. First of all, the WLF equation (Equations (4) and (5)) was replaced with the sigmoid function (Equations (6) and (7)). Furthermore, Equation (7) was used for nonlinear surface fitting to obtain two parameters (C_1_, C_2_). Finally, WLF equations and the parameters (C_1_, C_2_) were used for optimal fitting to get the single master curve.

(4) $$\log\left(a\left(T\right)\right)=\frac{-C_{1}\Delta T}{C_{2}+\Delta T}$$

(5) log(ξ)=log(ƒ)+ log(a(T)) 

where a(T) is the shifting factor at specific temperature T, ∆T is the temperature difference between the test temperature and the specified temperature. C_1_ and C_2_ are model constants. ξ and ƒ are the reduced frequency at the specified temperature and the test temperature, respectively.

(6) $$\log\left(G^{*}\right)=\delta +\frac{\alpha }{1+e^{\beta +\gamma \log\left(\xi \right)}}$$

(7) $$\log\left(G^{*}\right)=\delta +\frac{\alpha }{1+e^{\beta +\gamma \left(\log\left(ƒ\right)+\frac{-C_{1}\Delta T}{C_{2}+\Delta T}\right)}}$$

where β and γ are the shape parameters of the equation; α, δ is the span of G* values and the minimum modulus value, respectively.

Table 5 shows the parameters of the sigmoid function and WLF equation. In this Table, the column “Sigmoidal Function”, “R^2^@|G*|” means the determination coefficient of |G*| value.

**Figure 7:**
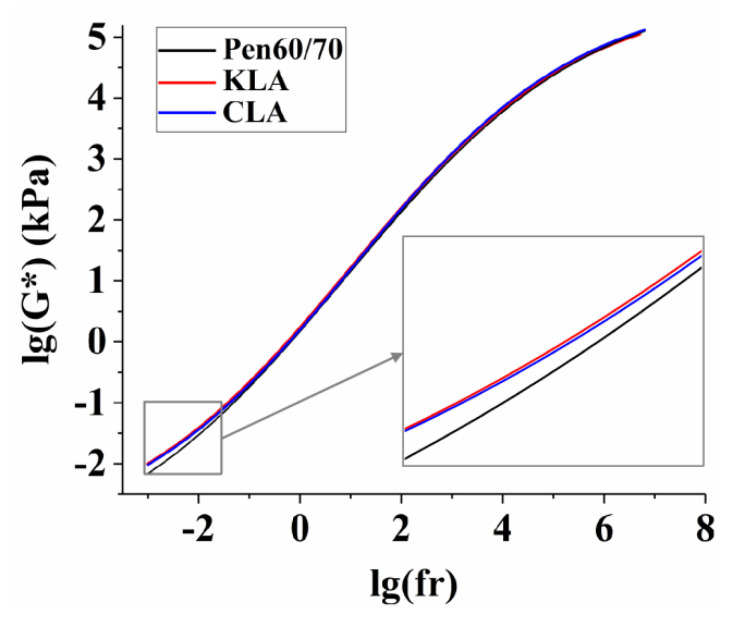
Master curves of test binders: Sigmoidal fitting curves.

The results of the target master curves of bitumen binders are shown in Figure 7. The sigmoidal fitting curves were obtained by the frequency sweep to value the overall rheological behavior of bitumen binders. As the principle of time-temperature superposition of viscoelastic materials indicates, high frequency is related to low temperature. As shown in the graph, it is clear that the increase in frequency led to the increase in modulus (G*). The G* values of LMA were higher in low frequencies compared to Pen60/70, but close in high frequencies, which reflected better performance in high temperatures, but it was not obvious. Meanwhile, the close results in high frequencies showed that the negligible effect in low-temperature performance of test binders with lignin. This finding supports past works conducted by Wang [47]. By comparison, lignin from wood chips had a slight improvement in high-temperature performance than that of lignin from corncobs. The results obtained from the main curves are consistent with the results of the MSCR test. 

### 3.2. Chemical Tests

#### 3.2.1. MWD (Molecular Weight Distribution)

**Figure 8:**
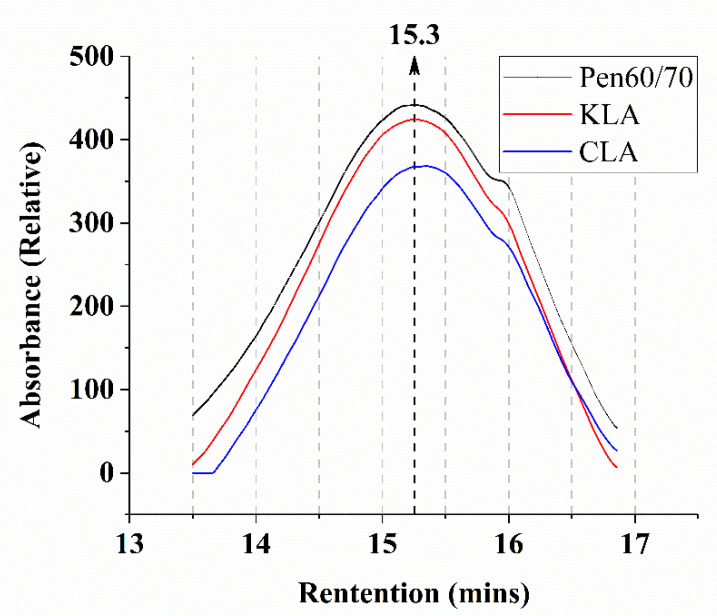
GPC chromatograms of three binders (Pen60/70, KLA, and CLA).

The gel permeation chromatography (GPC) test results of virgin bitumen are illustrated in Figure 8. As shown in Figure 8, the chromatogram is drawn mainly from 13 to 16.8 min of retention time, the retention time ranges from 13 to 16.8 min refers to Mw range is from 6076 to 272. As plotted, the main peak of each type of binder is mainly at 15.3 min and there is a small fluctuation of each test binder that occurred nearly 16 min.

**Figure 9:**
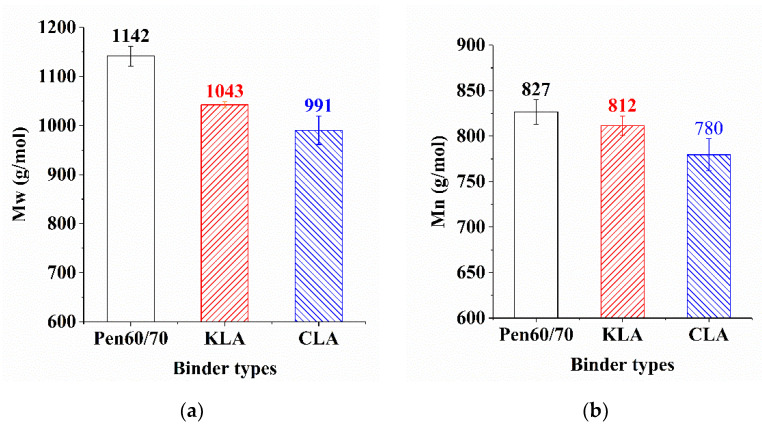
GPC test result: (**a**) weight-average molecular weight and (**b**) number-average molecular weight.

According to numerical statistics analysis, the GPC parameters are shown in Table 6 and Figure 9. To evaluate the molecular weight distribution of bitumen binders, five parameters including M_w_, M_n_, M_p_, M_z_, and PDI were selected for the statistical analysis of molecular weight distribution. Their meanings were listed as follows:

- M_n_ = number-average molecular weight (g/mol);
- M_p_ = peak molecular weight (g/mol);
- M_z_ = z-average molecular weight (g/mol);
- M_w_ = weight-average molecular weight (g/mol);
- PDI = M_w_/M_n_ = polydispersity Index (-).

In general, the larger the PDI, the broader the molecular weight [29]. As summarized in Table 6, Pen60/70 had the largest PDI value, indicating the broadest molecular weight, followed by KLA and CLA. Moreover, the M_w_ and M_n_ values are plotted in Figure 9a and b. It is apparent that KLA and CLA have lower molecular weight compared with Pen60/70 that is probably because the modification of KL and CL melts in bitumen fractions. The lignin with lower molecular weight melted in THF may be another reason for decreasing the molecular weight of the modified binder. CLA owned the lowest M_w_ and M_n_ values, therefore, the different sources of lignin led to the different molecular weight distributions of modified binders. In addition, the products of reaction between lignin as well as virgin binder may be insoluble in THF, which could be ascribed to the decrease of the molecular weight of bitumen binder. 

#### 3.2.2. Fourier-Transform Infrared Spectroscopy

**Figure 10:**
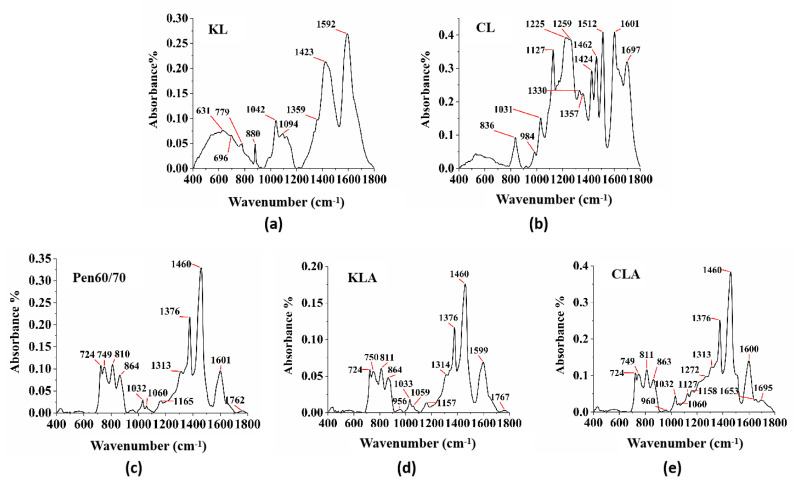
FTIR test results analysis: (**a**) KL; (**b**) CL; (**c**) Pen60/70; (**d**) KLA and (**e**) CLA.

FTIR was used as a probe to evaluate the chemical bonds and functional groups of lignin, binder, and lignin modified binder [48]. The FTIR results are shown in Figure 10. The fingerprint region between 400 and 1800 cm^−1^ wavenumbers were selected. The spectral analysis determined the spectral characteristics associated with the differences caused by lignin types [49]. Only KL showed an 880 cm^−1^ vibration correlated with guaiacyl lignin, while only CL showed 836 cm^−1^ vibration correlated with the C-H deformations asymmetric. In the CLA, there were other vibrations at 984, 1127 and 1462 cm^−1^ correlated with the C-H deformations asymmetric stretching [50]. CLA exhibited a more prominent the C-O group peak at nearly 1259 cm^−1^ and a stronger Stretching vibration of the C=O bond at 1697 cm^−1^ [46,49]. 

Figure 10 shows the absorption spectra of Pen60/70, KLA, and CLA. As shown in Figure 10c–e, it is obvious that the absorption spectra of KLA and CLA are similar to Pen60/70, but the peak area is different. These results show that each type of lignin is evenly distributed in bitumen. After lignin was added to virgin bitumen, there was no obvious chemical reaction occurred and KLA did not form different chemical bonds, while CLA had new functional groups. In the CLA, there are also obvious differences between regions the regions 1127 cm^−1^ and 1272 cm^−1^, for the conjugated C-O bond and C-H bond in syryngyl rings, respectively. A clear absorption at 1653 cm^−1^ was observed in the spectra of CLA, while that of KLA and Pen60/70 had no obvious absorption peak.

### 3.3. Mixture Test

#### 3.3.1. Marshall Test of Stability and Flow Value

**Figure 11:**
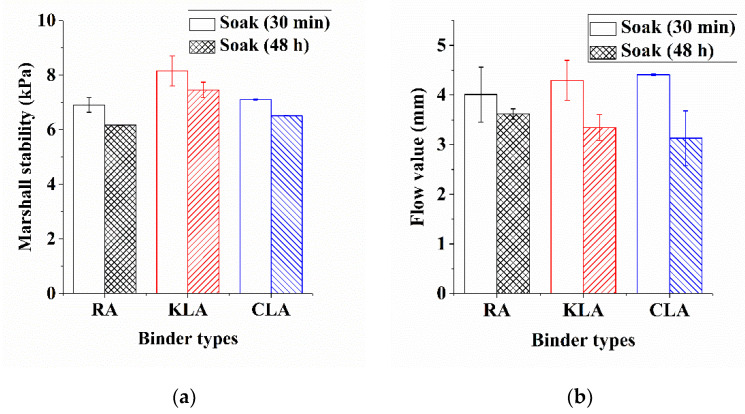
Marshall test results analysis: (**a**) Marshall stability and (**b**) flow value.

**Figure 12:**
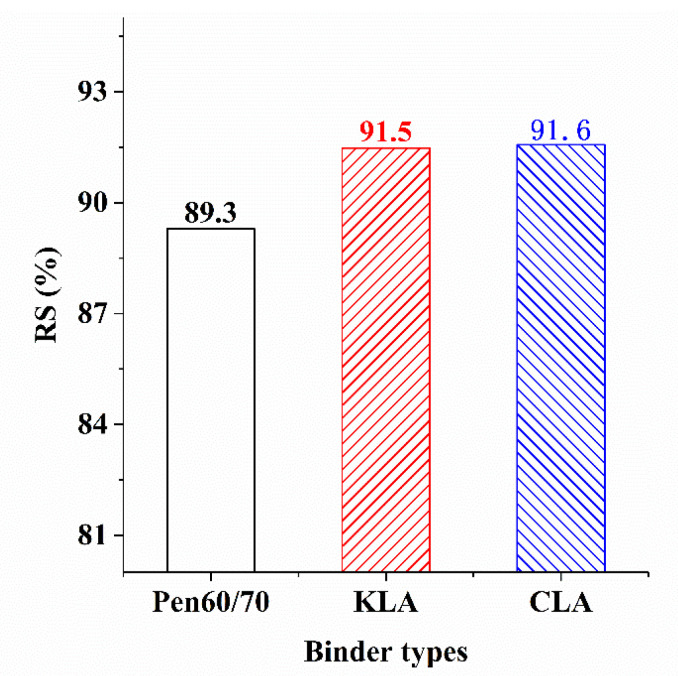
The RS of test samples.

As empirical indicators, Marshall Stability and flow value [51] were employed for quantifying the potential of bitumen mixture to permanent deformation. Marshall Stability estimated the maximum force that the mixture can withstand, and flow value evaluated the resistance of bitumen mixture to plastic deformation. The results of test specimens before and after 30 min or soaking in hot water for 48 h in a specific temperature (60 °C) are illustrated in Figure 11 and Figure 12 and Table 7. Figure 11a shows that mixtures with LMA own the higher Marshall Stability compared to mixtures with Pen60/70 in both soaking conditions. In 30 min soaking condition, the mixtures using KLA or CLA binders improved the Marshall Stability of the Pen60/70 mixture by 18.09% and 3.04%, respectively. In 48 h soaking condition, the Marshall Stability of the Pen60/70 mixture was improved by 20.91% and 5.67%, respectively, by the application of KL and CL. After soaking in hot water for 48 h, the flow value of mixtures using LMA was universally lower than Pen60/70, although the flow values of LMA mixtures after water soaking for 30 min were slightly higher than that of Pen60/70 mixtures, their differences were not significant. The higher Marshall Stability and the lower flow value is, the better performance at higher service temperatures the mixture has [47]. The Marshall stability and flow value test results indicate the positive effects on the permanent deformation resistance of Pen60/70 brought by lignin. Compared to CL, it can be concluded that KL further enhanced the positive effect on the high service temperature performance as its higher Marshall stability values. 

Residual Marshall Stability (RS) [52] refers to the ratio of Marshall Stability of the specimen to the virgin specimen after soaking in hot water for 48 h. The higher value of RS indicates better moisture stability [51]. As depicted in Figure 12, LMA mixtures are responsible for the higher RS values compared with the RS value of 89.3% of Pen60/70 mixture. KLA mixture and CLA mixture have similar RS values, 91.5%, and 91.6% respectively, indicating a better performance at resisting water damage. By comparison, The KL and CL further enhanced the positive effect on the water damage resistance by increasing RS value, 2.2% and 2.3% higher than that of Pen60/70. It can be observed that the difference among RS values of KLA and CLA were not obvious, the results show that the effect of lignin modifiers on the water damage resistance of mixture is basically same.

#### 3.3.2. Moisture Susceptibility

**Figure 13:**
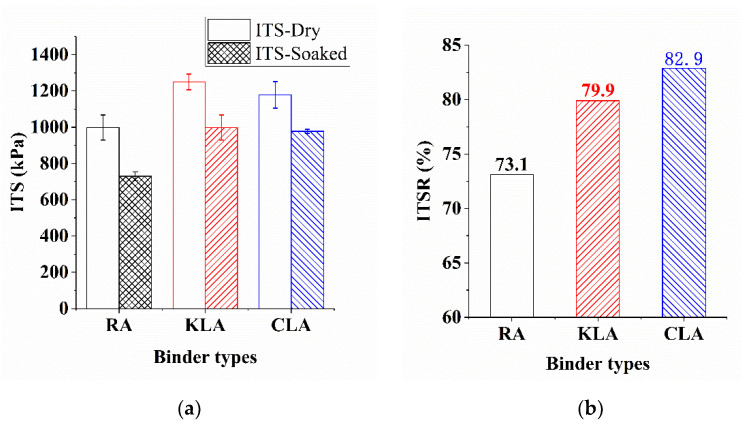
ITS test results analysis: (**a**) ITS values before and after a freeze-thaw cycle and (**b**) ITSR values.

ITSR refers to the ratio of the soaked specimens which had been through one freeze-thaw cycle to that of the original sample. Different from the RS method, ITSR evaluated the moisture susceptibility in hot and cold water condition. The ITS results of test specimens before and after the freeze-thaw cycle are plotted in Figure 13 and Table 8, It is apparent from the figures that LMA specimens possesses the higher ITS and ITSR in dry condition and freeze-thaw conditions. As shown in Figure 13a, in both conditions, KLA mixture was responsible for the highest ITS value, CLA mixture was responsible for secondary ITS value, followed by Pen60/70 mixture. The enhancement effect of the lignin modifiers was responsible for the higher ITS value of the LMA mixtures. The ITS test results indicate that lignin brought the positive effects on the indirect tensile strength both in hot and cold water condition. Moreover, it can be concluded that KL further enhanced the positive effect as its higher ITS values than those of CL. 

Figure 13b and Table 8 presents the ITSR values of test specimens, the test results of mixtures range from 73% to 83%. The ITSR values of all specimens were higher than the minimum value in the specification requirement. The ITSR values of LMA mixtures were 9–13% higher than that of Pen60/70 mixture, presenting a better performance of the moisture damage resistance in cold conditions. Besides, CLA mixture had the best moisture damage resistance, followed by KLA mixture and Pen60/70 mixture. Thus KL and CL further enhanced the moisture damage resistance in freezing condition due to their higher ITSR values than those of Pen60/70. It can be observed that the ITSR value of KLA was 79.9%, less than the 82.9% of CLA. However, the difference among ITSR values of KLA and CLA was not obvious. The ITSR results show that the effect of lignin modifiers on the frost damage resistance of mixture is basically similar.

#### 3.3.3. Aging Resistance and Modulus Stiffness

**Figure 14:**
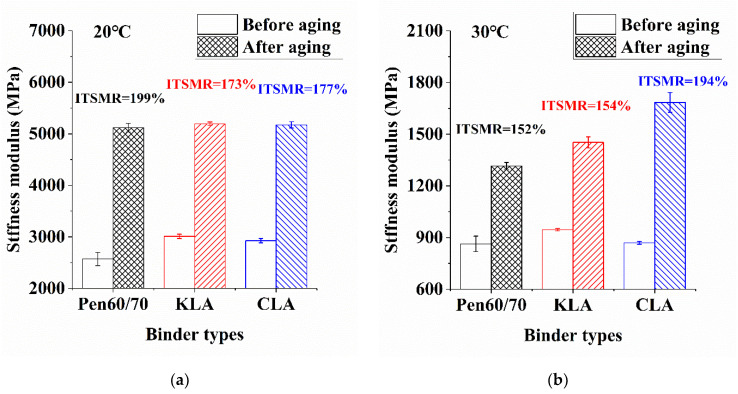
ITSM test results analysis: (**a**) before and after aging under 20 °C and (**b**) before and after aging under 30 °C.

Figure 14 shows the ITSM and ITSMR results before and after the long-term ageing. Before the long-term ageing, the ITSM values, in ascending order, were KLA, CLA and Pen60/70 mixture, both at 20 °C (a) and 30 °C (b). After the long-term ageing, the sequence of ITSM values followed the trajectory before aging process at 20 °C, but at 30 °C. It was observed that CLA mixture had the highest ITSM, followed by KLA and Pen60/70 mixtures. The ITSM test results show the enhancement of stiffness effect brought by lignin modifiers and aging procedure. At 20 °C, it can be seen that KL and CL slightly enhanced the stiffness as their ITSM values were similar with those of Pen60/70 after aging. However, at 30 °C, after aging, the enhanced effect on stiffness modules brought by CL was significant. CL had the largest ITSM value, showing the worst aging situation.

ISTMR is the ratio of ISTM after the ageing process divided by ISTM before the aging process. The value indicates the samples’ ageing sensitivity. As shown in Figure 14, the ISTMR values of Pen60/70, KLA, and CLA at 20 °C were 199%, 173%, and 177%, respectively. At 20 °C, LMA mixtures presented superior aging resistance than Pen60/70 mixture due to their lower ISTMR. Therefore, LMA mixtures may be performed better in colder climates. The ISTMR values of Pen60/70, KLA, and CLA at 30 °C were 152%, 154%, and 194%, respectively. The results show that Pen60/70 and KLA mixtures owned similar ITSMR values at 30 °C, indicating that KLA and Pen60/70 mixtures had similar aging resistance, and both exhibited better aging resistance than CLA mixture, which had the highest ITSMR value. Overall, it is found that the incorporation of KL improved the aging resistance of Pen60/70 mixture at 20 °C, while almost didn’t affect the aging resistance at 30 °C. To sum up, it is believed that KL is a better choice than CL considering the aging resistance of the mixture.

## 4. Conclusions

This paper evaluated the feasibility of lignin modification as performance improver for bituminous materials in details. A series of tests were performed on virgin and modified with lignin. The following findings can be obtained based on the test results:

- Lignin modified binder (5 wt.% of Pen60/70) showed the insignificantly improved high-temperature performance and low-temperature performance than virgin bitumen binder (Pen60/70).
- The lignin modification improved the viscosity, stiffness, soften point, rutting resistance, and elastic recovery of virgin binder (Pen60/70). However, lignin had slightly negative effects on the fatigue resistance, and reduced the fatigue life of the bitumen binder.
- Bituminous mixture with 5% lignin improved the permanent deformation resistance, moisture susceptibility, and aging resistance. LMA mixtures outperformed bitumen binder (Pen60/70) mixture in low temperature.
- The FTIR results indicate that the application of lignin did not remarkably change functional groups of bitumen binder. Lignin has different chemical bonds depending on lignin sources. KL showed an 880 cm^−1^ vibration correlated with guaiacyl, while CL showed 1697 cm^−1^ vibration correlated with carbonyl. The GPC results show that the application of lignin decreased the molecular weight of asphalt binder.
- KL had better improvements in rutting resistance of binder, permanent deformation resistance and aging resistance of mixture than CL. However, CL was slightly better at improving the workability and low-temperature performance of mixture. Overall, the Kraft lignin derived from wood chips showed superior performance in bitumen modification than that extracted from corn stalk residue.

Finally, this research provided a more comprehensive understanding of the lignin modification as a performance enhanced for bituminous materials. Future study will be focused on the thermal characteristics, the in-situ validation and life cycle assessment of bituminous pavement with different lignin modifiers.

## Figures and Tables

**Table 1:** Properties of KL (Kraft lignin) and CL (corn stalk lignin).

	KL	CL
Feature	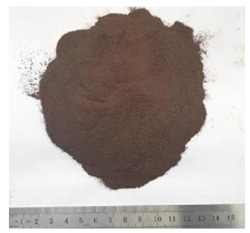	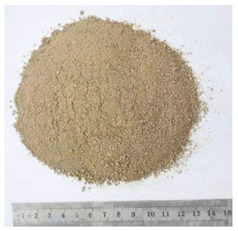
SEM	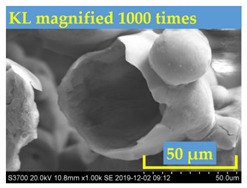	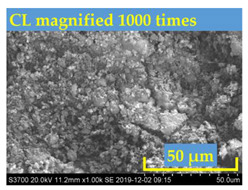
Source	Aspen wood chips	Corn stalk residues
Production Place	Nanjing China	Jinan China
Diameter	Less than 0.15 mm	Less than 0.15 mm
PH Value	8	7–8
Ash Content (by weight)	1.3%	Less than 1%
Water Content (by weight)	5%	Less than 5%
Dry Mass Content (by weight)	95%	More than 90%

**Table 2:** Design gradation information.

BS Sieve Size	Percent Passing by Mass (%)	Passing Requirement (%)
14 mm	100	100
10 mm	96	92–100
5 mm	35	28–42
2.36 mm	26	19–33
75 um	9.8	7.8–11.8 (including 2% hydrated lime)

**Table 3:** MSCR test results (The numbers after “±” are standard deviations).

Binder Types	% Recovery		J_nr_		
	0.1 kPa (kPa^−1^)	3.2 kPa (kPa^−1^)	0.1 kPa (kPa^−1^)	3.2 kPa (kPa^−1^)	J_nr-diff_
Pen60/70	0.400 ± 0.350	0.000 ± 0.000	2.578 ± 0.070	2.766 ± 0.056	7.300 ± 0.700
KLA	1.350 ± 0.050	0.100 ± 0.000	2.366 ± 0.041	2.552 ± 0.046	7.850 ± 0.050
CLA	1.050 ± 0.050	0.200 ± 0.000	2.025 ± 0.011	2.180 ± 0.010	7.650 ± 0.050

**Table 4:** BBR test result analysis.

Binder Types	−6 °C		−12 °C		−18 °C	
	Stiffness (MPa)	m-Value (×10^−2^)	Stiffness (MPa)	m-Value (×10^−2^)	Stiffness (MPa)	m-Value (×10^−2^)
Pen60/70	156	34	284	31	441	21
KLA	142	35.9	233	32.5	369	24.5
CLA	132	37.1	226	38	378	29

**Table 5:** Model parameters.

Parameters	WLF Equation		Sigmoidal Function				
	C_1_(-)	C_2_(-)	δ(Pa)	α(Pa)	β(-)	γ (-)	R^2^@|G*|
Pen60/70	13.88	191.7	−3.64	9.189	−0.2065	−0.4195	0.9988
KLA	11.78	176.4	−4.861	10.83	−0.1006	−0.3637	0.9987
CLA	15.82	222.1	−3.304	9.071	−0.4636	−0.445	0.9978

**Table 6:** GPC parameters (The numbers after “±” are standard deviations).

Sample ID	M_n_ (g/mol)	M_p_ (g/mol)	M_z_ (g/mol)	M_w_ (g/mol)	PDI (-)
Pen60/70	827 ± 13	920 ± 13	1603 ± 111	1142 ± 21	1.38151 ± 0.0473
KLA	812 ± 11	903 ± 10	1326 ± 2	1043 ± 6	1.28472 ± 0.0089
CLA	780 ± 18	873 ± 26	1241 ± 48	991 ± 29	1.27060 ± 0.0084

**Table 7:** Marshall stability and flow value test analysis (The numbers after “±” are standard deviations).

Binder Types	Strength (kPa)		RS (%)	Flow Values (mm)	
	Soak (30 min)	Soak (48 h)		Soak (30 min)	Soak (48 h)
Pen60/70	6.91 ± 0.27	6.17 ± 0.01	89.3	4.01 ± 0.55	3.62 ± 0.11
KLA	8.16 ± 0.54	7.46 ± 0.28	91.5	4.30 ± 0.40	3.35 ± 0.26
CLA	7.12 ± 0.02	6.52 ± 0.01	91.6	4.41 ± 0.01	3.13 ± 0.55

**Table 8:** The ITS and ITSR values (The numbers after “±” are standard deviations).

Binder Types	Freeze Samples (kPa)	ITSR (%)	Dry Samples (kPa)
Pen60/70	729.8 ± 19.7	73.1	998.2 ± 56.5
KLA	998.1 ± 56.6	79.9	1249.6 ± 35.2
CLA	977.3 ± 9.0	82.9	1178.3 ± 59.9

## Data Availability

The data presented in this study are available on request from the corresponding author.
